# Unmet cancer rehabilitation needs and access to survivorship services across the cancer continuum

**DOI:** 10.1007/s00520-026-10752-5

**Published:** 2026-05-11

**Authors:** Emer Guinan, Naomi Algeo, Arún Fenton, Juliet Herbst, Caoimhe Mulgrew, Gráinne Sheill, Ganna Eissa, Ailish Hanrahan, Catherine O’Brien, Sarah Wade, Alison Enright, M. John Kennedy

**Affiliations:** 1 School of Medicine, Trinity College Dublin, Dublin, Ireland; 2 Trinity St James’s Cancer Institute, Dublin, Ireland; 3 Department of Occupational Therapy, St James’s Hospital, Dublin, Ireland; 4 Department of Clinical Nutrition, St James’s Hospital, Dublin, Ireland; 5 Department of Medical Social Work, St James’s Hospital, Dublin, Ireland; 6 Department of Speech and Language Therapy, St James’s Hospital, Dublin, Ireland; 7 Department of Physiotherapy, St James’s Hospital, Dublin, Ireland; 8 The Haematology, Oncology and Palliative Care (HOPe) Directorate, St James’s Hospital, Dublin, Ireland; 9 Health and Social Care (SCOPe) Directorate, St James’s Hospital, Dublin, Ireland; 10 Discipline of Physiotherapy, Trinity Centre for Health Sciences, St James’s Hospital, Dublin, Ireland

**Keywords:** Cancer rehabilitation, Allied health professional, Health needs assessment, Cancer survivorship, Quality of life

## Abstract

**Purpose:**

To investigate perceived rehabilitation needs and service utilisation amongst people living with and beyond cancer in a large Comprehensive Cancer Centre in Ireland and examine associations between impairment and demographic and clinical parameters.

**Methods:**

Utilising a prospective, observational design, a consecutive sample of participants attending outpatient clinics was enrolled in June and July 2025. Demographic data, clinical data, and use of allied health professional (AHP) cancer rehabilitation services were self-reported. Unmet needs were captured using the Macmillan Holistic Needs Assessment. Data were analysed using descriptive statistics and multivariate regression models.

**Results:**

In total, 660 surveys were analysed. Most participants were female (58.2%) and aged between 56 and 75 years (54%). Haematological cancers (25.6%) and breast cancers (22.9%) were the two largest cohorts, and most patients (57%) were currently on treatment. Prevalence of AHP cancer rehabilitation needs was considerable with an average of 71% of patients reporting at least one specialist need and 49% reporting at least two specialist needs across professions. On treatment status was consistently associated with greater odds of patients reporting specialised needs. However, only 36% of patients with perceived needs reported seeing a relevant AHP since their diagnosis.

**Conclusions:**

Results confirm that patient-reported rehabilitation needs are significant across the cancer trajectory, however utilisation of cancer rehabilitation AHPs is suboptimal.

**Implications for cancer survivors:**

Strategic development of the cancer rehabilitation AHP workforce and service models is required to mitigate the impact of cancer and its treatment on functional, nutritional, and psychosocial patient outcomes, and optimise health-related quality of life.

**Supplementary Information:**

The online version contains supplementary material available at 10.1007/s00520-026-10752-5.

## Introduction

As survival rates for many cancers improve, an increasing number of people live with and beyond cancer, yet they often face a wide range of physical, psychosocial, and functional consequences of both the disease and its treatment [1]. These may include fatigue, pain, neuropathy, decreased mobility, lymphoedema, cognitive changes, emotional distress, and difficulty returning to normal daily activities or work [2]. Multidisciplinary outpatient cancer rehabilitation can improve patients’ physical and psychosocial status [3]. Allied health professionals (AHPs) are a specialised workforce that bring unique profession-specific skills, knowledge, and competencies to address distinct cancer-related impairments [4–8], and who provide evidence-based interventions within the domain of cancer rehabilitation that optimise function, nutrition, psychosocial wellbeing, and quality of life [9].

Systematic assessment and documentation of rehabilitation needs at key points (such as during and following treatment) allows for timely identification of impairments, activity limitations, and participation restrictions [10]. Guidance from the National Institute for Health and Care Excellence (NICE) emphasises that rehabilitation needs should be assessed throughout the cancer pathway [11]. Documenting the perceived rehabilitation needs of people during or after treatment for cancer may support better service design, resource allocation, and policy development for multiple professions and facilitate more consistent standards of care [12]. It also may support survivors to engage with services that support return to social, occupational, and everyday life.

While global estimates highlight the growing demand for cancer rehabilitation internationally, rehabilitation needs can vary widely between countries due to differences in disease prevalence, ageing populations, health infrastructure, and socioeconomic conditions. Several population-based studies from the USA have reported an increased burden of physical performance limitations and participation restrictions amongst individuals living with and beyond cancer compared to those without a cancer history [13–17]; however, these studies are typically restricted to physical domains of cancer rehabilitation, with limited consideration of psychological, cognitive, or social domains. Accordingly, there is a need to document this information in an Irish population, across multiple dimensions of cancer rehabilitation and with consideration of multiple members of the cancer rehabilitation workforce [18–20]. Also, as rehabilitation needs may vary by cancer type, treatment modality, and individual patient factors (age, gender), there is a need to explore factors that may be associated with a greater need for support.

Ireland has witnessed significant advancements in cancer survivorship over the past decade, underpinned by a commitment in the 2017–2026 National Cancer Strategy to maximise patient involvement and the quality of life of those living with and beyond cancer (Goal 4.4), reflecting for the first time a national commitment to survivorship care [21]. Under this strategy, the cancer survivorship programme at the National Cancer Control Programme has published several models of care, including frameworks for stratified self-management follow-up [22] and psycho-oncology [23], which have informed practice standards and workforce provision. Nationally, however, a major gap remains in the provision of AHP-delivered services, with no model of care to inform future directions. With a new National Cancer Strategy due for development in 2026/2027, it is timely to conduct a contemporary needs assessment within the context of the only national Comprehensive Cancer Centre, to understand current demand in order to plan strategic workforce investment and service development models, including referral pathways, resource allocation, and models of care.

The overall aim was to investigate unmet rehabilitation needs and access to AHP rehabilitation services in people living with and beyond cancer in a large Comprehensive Cancer Centre in Ireland.

The specific objectives were:

1. To describe the prevalence of perceived rehabilitation needs across the cancer care trajectory
2. To describe the use of AHP rehabilitation services since cancer diagnosis
3. To examine associations between perceived rehabilitation needs and demographic and clinical parameters

## Methods

### Study design

Utilising a prospective, observational design, a consecutive sample of patients with a diagnosis of cancer attending surgical oncology, medical oncology, and haematology outpatient clinics at the Trinity St James’s Cancer Institute (TSJCI) Comprehensive Cancer Centre were enrolled in June and July 2025. All patients with a confirmed diagnosis of cancer, across all cancer stage and treatment trajectories, were eligible to participate. Eligibility status was confirmed in consultation with the treating medical and nursing teams. Data were entered prospectively and monitored weekly for demographic representation. The sampling strategy was adapted accordingly to target clinics with higher volumes of underrepresented cancers and age profiles. Participation was voluntary, and consent was implied through anonymous survey return. Ethical approval was obtained from the Tallaght University Hospital/St James’s Hospital Joint Research Ethics Committee (Project ID 4660). This manuscript adheres to the STROBE reporting guidelines [24].

### Data collection and survey instrument

Patient-reported data were collected using paper-based and electronic survey options. Demographic (age, gender) and clinical (cancer diagnosis, stage, and treatment history) data were self-reported. Use of AHP cancer rehabilitation services, including physiotherapy, occupational therapy, speech and language therapy, dietetics, or medical social work, at any point since cancer diagnosis, was self-reported as yes, no, or unsure. The AHP cancer rehabilitation services examined align with international cancer rehabilitation workforce guidance [9, 25] and are relevant to the context of the health system within our Comprehensive Cancer Centre, where all five disciplines are governed within one Clinical Directorate.

Unmet needs were captured using the Macmillan Holistic Needs Assessment (HNA) available online [26]. This questionnaire checklist comprises 72 needs categorised into six domains: physical (*n* = 27 needs), practical (*n* = 16 needs), emotional (*n* = 12 needs), family or relationship (*n* = 5 needs), spiritual (*n* = 3 needs), and information or support (*n* = 9 needs). Respondents identify needs that apply to them (scored 1). Needs that are not relevant are not selected (scored 0). Only the presence or absence of a need, and not the severity of that need, was measured. One open-text question at the end of the questionnaire asked participants to write which three needs from the 72-item list were most important to them. The final survey instrument, incorporating demographic, clinical, and service use questions, in addition to the Macmillan HNA, was reviewed by the TSJCI Patient Representative Group and formatted to maximise readability in advance of distribution. Potential participants were invited to complete the anonymous survey (paper-based or online) while waiting for their clinical consultation at a routine outpatient clinic visit, or alternatively, to complete the survey within 1 week of their appointment online. Double data entry procedures were used for paper-based surveys. English versions of the survey tool were professionally translated to Russian, Polish, Ukrainian, and Romanian, the top four languages utilised by hospital interpretation services.

### Statistical analysis

Statistical analyses were performed using IBM SPSS (Statistical Package for the Social Sciences) Statistics software, version 28. Data normality was assessed graphically using histograms and Kolmogorov–Smirnov tests. Descriptive statistics were used for demographic and cancer variables, AHP cancer rehabilitation service utilisation, and the presence or absence of concerns across each domain. Summary statistics for continuous variables (means and standard deviations or medians and ranges as appropriate) and categorical variables (counts and proportions) were calculated, with subgroup analyses as appropriate. Univariate analysis of total concerns data across demographic and cancer variables was performed using non-parametric Kruskal–Wallis tests and chi-square tests as appropriate. Open-text responses were analysed using content analysis and expressed as citation frequency.

Concerns data were mapped to each profession and analysed as specialist AHP cancer rehabilitation needs relevant to each profession. Mapping was completed by a multidisciplinary team of Clinical Specialist and Senior AHPs specialising in cancer rehabilitation and underpinned by relevant literature [4–8], and reflected current service provision, professional roles, and referral pathways within our Comprehensive Cancer Centre (Supplemental File 1). Univariate and multivariable logistic regression models were used to examine factors associated with having any specialist AHP rehabilitation need specific to each profession. Covariates in all models included age groups, gender, cancer type, cancer stage, and treatment status. All covariates were treated as categorical variables using dummy (indicator) variable coding systems. Reference values were consistent across all models, as indicated in Table 4. The underlying assumptions of logistic regression were tested and confirmed using the Omnibus Test of Model Coefficients and the Hosmer–Lemeshow assessment.

## Results

Between June 12 and July 4, 2025 (16 days), 27 outpatient clinics including surgical oncology (*n* = 8), medical oncology (*n* = 8), haematology (*n* = 9), and special populations (e.g. geriatric oncology clinic, adolescent and young adult clinic) (*n* = 2) clinics were attended at least twice. During this period, 750 questionnaires were distributed, and 677 were returned. Of the completed questionnaires, *n* = 10 were excluded due to incomplete demographic details and *n* = 7 were excluded due to a non-cancer diagnosis (e.g. patients treated with stem cell transplant for a non-cancer condition), leaving a final sample of *n* = 660 completed questionnaires (*n* = 632 paper copies and *n* = 28 online), representing an 88% recruitment rate. Four questionnaires were distributed in non-English languages, and two were returned. Demographic and clinical characteristics of the sample are presented in Table 1.

**Table 1:** Demographics and cancer characteristics

Characteristic	*n* (%)
Age range	
<25 years	31 (4.7)
26–45 years	79 (12)
46–55 years	105 (15.9)
56–65 years	182 (27.6)
66–75 years	174 (26.4)
>75 years	89 (13.5)
Gender	
Female	384 (58.2)
Male	276 (41.8)
Cancer type	
Breast	151 (22.9)
Urological	40 (6.1)
Lung	47 (7.1)
Colorectal	34 (5.2)
Gynaecological	64 (9.7)
Head and neck	72 (10.9)
Haematological	169 (25.6)
Upper GI	63 (9.5)
Other	20 (3.0)
Stage	
Stage I–III (curative)	227 (34.5)
Stage IV/advanced	196 (29.7)
Don’t know	237 (35.9)
Treatment status	
Currently on treatment	376 (57)
Finished treatment in the last year	114 (17.3)
Finished treatment >1 year ago	100 (15.2)
None/awaiting plan	60 (9.1)
Not answered	10 (1.5)
Treatments received*	
None/awaiting plan	35 (5.3)
Surgery	246 (37.3)
Radiation therapy	263 (39.8)
Immunotherapy	134 (20.3)
Bone marrow transplant	67 (10.2)
Chemotherapy	423 (64.1)
Hormone therapy	78 (11.8)
Other	48 (7.3)
Number of treatment modalities	
0	29 (4.4)
1–2	435 (65.9)
3–4	181 (27.4)
5+	6 (0.9)
Not answered	6 (0.9)

Data are presented as frequency (percentage) within each category. *Total percentage >100% as patients were treated with multiple treatment modalities

The majority of participants were female (58.2%) and were aged between either 46 and 55 years (15.9%), 56 and 65 years (27.6%), or 66 and 75 years (26.4%). Eight different cancer types were represented, with haematological cancers (25.6%) and breast cancers (22.9%) representing the two largest cohorts. Almost one third of the cohort (29.7%) reported having stage IV/advanced cancer, one third did not know their cancer stage (35.9%), and the remainder reported a diagnosis of stage I/II/III disease. The majority of patients (57%) were currently receiving treatment. Participants reported receiving multiple different treatment modalities including chemotherapy (64.1%), radiation therapy (39.8%), surgery (37.3%), and immunotherapy (20.3%). Most participants received more than one treatment modality.

### Needs assessment

Participants reported a median of 11 (IQR 3) concerns in total on the needs assessment. The most commonly reported concerns were tired, exhausted, or fatigued (*n* = 400, 60.61%); exercise and activity (*n* = 265, 40.15%); thinking about the future (*n* = 260, 39.39%); worry, fear, or anxiety (*n* = 245, 37.12%); and uncertainty (*n* = 236, 35.76%). Content analysis of the needs that participants identified as the most important to them identified health and wellbeing (*n* = 129), exercise and activity (*n* = 107), managing my symptoms (*n* = 105), and diet and nutrition (*n* = 103) as the most frequently cited (Table 2).

**Table 2:** Most important needs to participants (top 10)

What is most important to you?	Frequency
Health and wellbeing	129
Exercise and activity	107
Managing my symptoms	105
Diet and nutrition	103
Planning for my future priorities	66
Tired, exhausted, or fatigued	59
Uncertainty	31
Worry, fear, or anxiety	27
Thinking of the future	24
Patient or care support groups	22

Data are presented as the 10 most frequently cited needs that are most important to participants across the entire sample

### Prevalence of rehabilitation need and use of AHP cancer rehabilitation services

Prevalence of AHP cancer rehabilitation needs was considerable, with an average of 71% of patients reporting at least one specialist need and 49% reporting at least two specialist needs. This varied across professions as outlined in Table 3. Specialist needs were mapped to a history of relevant AHP cancer rehabilitation service input. Across all five professions, an average of 36% of patients with perceived rehabilitation needs reported seeing a relevant AHP cancer rehabilitation professional since diagnosis (Table 3). This varied considerably across professions:

- 19% of patients with perceived occupational therapy needs reported accessing occupational therapy services.
- 31.5% of patients with perceived medical social work needs reported accessing medical social worker services.
- 38.5% of patients with perceived speech and language therapy needs reported accessing speech and language therapy services.
- 43.2% of patients with perceived physiotherapy needs reported accessing physiotherapy services.
- 48.2% of patients with perceived dietetics needs reported accessing dietetics services.

**Table 3:** Cancer rehabilitation needs across professions

	Physiotherapy	Occupational therapy	Speech and language therapy	Dietetics	Medical social work
At least one need potentially requiring input	456 (69.1)	523 (79.2)	263 (39.8)	555 (84.1)	547 (82.9)
At least two needs potentially requiring AHP input	273 (41.4)	368 (55.8)	92 (13.9)	422 (63.9)	469 (71.1)
Overall burden					
0	204 (30.9)	137 (20.8)	397 (60.2)	105 (15.9)	113 (17.1)
1	183 (27.7)	155 (23.5)	171 (25.9)	133 (20.2)	78 (11.8)
2	120 (18.2)	122 (18.5)	57 (8.6)	103 (15.6)	55 (8.3)
3	85 (12.9)	95 (14.4)	24 (3.6)	94 (14.2)	56 (8.5)
4	40 (6.1)	63 (9.5)	11 (1.7)	69 (10.5)	47 (7.1)
5–10	28 (4.3)	88 (13.3)	0 (0)	153 (23.2)	200 (30.3)
>10	0 (0)	0 (0)	0 (0)	3 (0.5)	111 (17.1)

Prevalence data are presented as frequency (percentage)

### Factors associated with profession-specific rehabilitation needs

On multivariable logistic regression analysis, patients reporting physiotherapy needs were more likely to be younger (age 26–45 years, OR 3.15, 95% CI 1.14, 8.71; age 46–55 years, OR 2.7, 95% CI 1.09, 6.64) than those aged >75 years and were more likely to be on treatment (OR 2.37, 95% CI 1.19–4.70) (Table 4). Similarly, patients reporting occupational therapy needs were more likely to be aged 26–45 years and aged >75 years (OR 2.94, 95% CI 1.11, 7.83), and currently on treatment (OR 4.44, 95% CI 2.33, 8.45), but were less likely to have urological cancer than breast cancer (OR 0.37, 95% CI 0.14, 0.98) (Table 4).

**Table 4:** Factors associated with profession-specific rehabilitation needs

	Physiotherapy			Occupational therapy			Speech and language therapy			Dietetics			MSW		
	OR	95% CI	*p* value	OR	95% CI	*p* value	OR	95% CI	*p* value	OR	95% CI	*p* value	OR	95% CI	*p* value
Age range															
<25	0.57	0.2–16.1	0.29	3.31	0.84–13.11	0.09	0.87	0.34–2.26	0.78	1.46	0.45–4.81	0.53	**	**	**
26–45	3.15	1.1–16.8	0.03	2.94	1.11–7.83	0.03	1.19	0.60–2.35	0.61	2.02	0.80–5.08	0.14	1.54	0.64–3.69	0.33
46–55	2.70	1.1–16.6	0.03	1.89	0.82–4.36	0.14	0.88	0.47–1.66	0.69	1.57	0.68–3.60	0.29	2.27	0.96–5.35	0.06
56–65	1.02	0.5–16.2	0.96	0.96	0.49–1.88	0.91	1.02	0.57–1.81	0.95	1.37	0.65–2.85	0.41	1.04	0.55–1.96	0.91
66–75	1.19	0.6–16.2	0.61	0.86	0.45–1.63	0.63	0.65	0.36–1.16	0.14	1.10	0.55–2.19	0.79	0.60	0.33–1.09	0.09
>75	Ref			Ref			Ref			Ref	–		Ref	–	
Gender															
Female	0.98	0.57–1.7	0.94	0.98	0.58–1.65	0.95	0.91	0.60−0.139	0.68	1.16	0.64–2.11	0.63	2.18	1.27–3.75	0.01
Male	Ref			Ref			Ref			Ref			Ref		
Cancer type															
Urological	0.78	0.3–2.2	0.64	0.37	0.1–1.0	0.04	0.36	0.1–1.0	0.05	0.75	0.3–2.1	0.58	1.81	0.6–5.4	0.29
Lung	1.08	0.4–2.8	0.87	0.74	0.3–1.8	0.52	0.87	0.4–1.9	0.72	0.74	0.3–1.8	0.50	0.59	0.2–1.4	0.23
Colorectal	0.99	0.3–3.1	0.99	0.84	0.3–2.5	0.76	1.50	0.7–3.4	0.34	2.24	0.6–8.5	0.24	1.29	0.4–4.2	0.67
Gynaecological	1.19	0.5–2.8	0.69	1.38	0.6–3.3	0.47	1.28	0.7–2.4	0.44	1.25	0.6–2.8	0.59	0.65	0.3–1.4	0.29
Head and neck	0.97	0.4–2.3	0.94	1.06	0.5–2.4	0.89	4.97	2.5–10.0	<0.001	3.34	1.2–9.3	0.02	1.38	0.6–3.3	0.47
Haematological	1.61	0.7–3.6	0.24	1.35	0.6–2.8	0.44	1.26	0.7–2.2	0.44	1.96	0.9–4.3	0.09	1.23	0.6–2.6	0.60
Upper GI	1.10	0.4–2.7	0.84	0.91	0.4–2.2	0.84	3.77	1.9–7.6	<0.001	4.19	1.2–14.1	0.02	1.63	0.6–4.2	0.31
Other	0.57	0.2–2.0	0.38	0.38	0.1–1.3	0.11	0.40	0.1–1.5	0.17	0.71	0.2–2.5	0.59	2.63	0.5–13.8	0.25
Breast	Ref			Ref			Ref			Ref			Ref		
Stage															
Stage I/II/III	0.70	0.41–1.2	0.20	0.98	0.58–1.66	0.95	0.98	0.63–1.51	0.92	0.91	0.52–1.60	0.73	1.15	0.69–1.93	0.60
Stage IV/advanced	1.10	0.6–2.02	0.76	1.22	0.69–2.16	0.49	1.05	0.67–1.65	0.83	1.14	0.62–2.13	0.67	1.49	0.85–2.62	0.16
Don’t know	Ref			Ref			Ref			Ref			Ref		
Treatment status															
Currently on treatment	2.37	1.20–4.70	0.01	4.44	2.33–8.45	<0.001	2.25	1.17–4.35	0.02	2.54	1.06–6.05	0.04	2.06	1.05–4.05	0.04
Finished treatment in the last year	1.66	0.75–3.68	0.21	1.56	0.76–3.19	0.23	1.43	0.68–3.00	0.34	1.49	0.57–3.85	0.42	1.45	0.67–3.12	0.35
Finished treatment >1 year ago	1.34	0.62–2.90	0.46	1.85	0.89–3.85	0.10	1.47	0.69–3.12	0.32	1.03	0.38–2.82	0.95	1.53	0.70–3.33	0.29
None/awaiting plan	Ref			Ref			Ref			Ref			Ref		

Logistic regression models of age range, gender, cancer type, stage, and treatment status on the presence or absence of at least one profession-specific need. The dependent variables for each analysis were binary (0/1), indicating the presence or absence of at least one profession-specific need across each of the following professions: 1. Physiotherapy-specific need, 2. Occupational therapy-specific need, 3. Speech and language therapy-specific need, 4. Dietetics-specific need, 5. Medical social work-specific need. Covariate selection was decided a priori in line with demographics collected and the same covariates were applied in all models

*GI* gastrointestinal, *OR* odds radio, *CI* confidence interval

**Odds ratio could not be calculated as all 31 participants aged <25 indicated as least one rehabilitation need that required medical social work input

Patients reporting speech and language therapy needs were more likely to be on treatment (OR 2.25, 95% CI 1.17, 4.35) and have head and neck cancer (OR 4.97, 2.46, 10.04) or upper gastrointestinal cancer (OR 3.77, 95% CI 1.87, 7.60) compared to breast cancer, but were less likely to have urological cancer (OR 0.36, 95% CI 0.13, 0.99) (Table 4). Similarly, patients reporting dietetics needs were more likely to be on treatment (3.59, 95% CI 1.81, 7.10) and to have a diagnosis of head and neck cancer (OR 3.34, 95% CI 1.20, 9.26) or upper gastrointestinal cancer (OR 4.19, 95% CI 1.25, 14.08) compared to breast cancer. Finally, patients reporting medical social work needs were more likely to be <25 years (all patients) or aged 46–55 (OR 3.49, 95% CI 1.45, 8.38) than aged >75 years, female rather than male (OR 2.18, 95% CI 1.26, 3.77), and on treatment (OR 2.01, 95% CI 1.02, 3.97).

## Discussion

Results confirm that the prevalence of perceived cancer rehabilitation needs is high, with up to 84% of patients reporting at least one rehabilitation need that could be addressed by AHP cancer rehabilitation services and up to 71% reporting at least two specialist rehabilitation needs. This study provides a unique characterisation of cancer rehabilitation needs in the context of AHP cancer rehabilitation service specialisation and utilisation in a large cohort attending surgical oncology, medical oncology, haematology, and specialist outpatient clinics at a high-volume, comprehensive cancer centre. An average of 36% of patients with identified rehabilitation needs reported seeing a relevant AHP since their diagnosis. In adjusted models, on treatment status was consistently associated with greater odds of patients reporting specialised needs across all professions.

In this cohort, the most reported concerns were tired, exhausted, or fatigued; exercise and activity; thinking about the future; worry, fear, or anxiety; and uncertainty. When adjusting for demographic and clinical factors, younger age, female gender, and on treatment status were associated with a higher number of total reported concerns. Fatigue is one of the most universal symptoms reported by patients with cancer, with an average 43% prevalence across all cancers and treatment stages, with the highest levels of *severe fatigue* reported in patients who are on treatment, female gender, and with certain tumour types [27]. Similarly, moderate levels of fear of cancer recurrence are reported in 59% of cancer survivors, with the highest levels in patients of younger age and female gender [28]. The benefits of exercise and activity across the cancer trajectory are now well described [29, 30], and efforts are underway to support implementation into practice [31]; however, treatment side effects, including fatigue, are key barriers to exercise participation and patient education is often insufficient to overcome patient concerns [32], as reflected here by patients highlighting exercise and activity as a significant information need.

While patient-reported needs were diverse and consistent with the wider literature, the distinctive component of this analysis was to examine unmet needs in the context of AHP skills, expertise, and past service utilisation. In this analysis, the first in Ireland, most participants had at least one reported need that would have been amenable to either physiotherapy or occupational therapy (69% and 79%, respectively). These data are consistent with data from the USA from Pergolotti et al. who reported that 65% of older adults had at least one physiotherapy/occupational therapy rehabilitation deficit [16], and Cheville et al. who reported at least one physical impairment in 92% of patients with metastatic breast cancer [33]. As an indicator of cumulative burden in our sample, 41% reported at least two physiotherapy impairments and 56% reported at least two occupational therapy-specific impairments, findings which are consistent with Flores et al. who reported moderate-to-severe physical functional impairment in 40% of their sample of patients who similarly spanned the cancer trajectory [15]. Pergolotti et al. reported that only 9% of patients with identified needs had received physiotherapy or occupational therapy within the previous 12 months [16]. In our sample, reported use of physiotherapy or occupational therapy since diagnosis amongst patients with identified needs was higher (43% and 19%), but access remains suboptimal. Unmet dietetic needs were the most reported issue in our sample, with 84% reporting at least one dietetic need and 64% reporting at least two specialised needs. These data suggest a significantly higher burden of need in comparison to a recent national survey in which 45% of patients reported diet-related problems and 44% reported weight loss [34]. In our analysis, only half (48%) of patients with identified dietetic needs reported seeing a dietitian within the past 12 months, which is consistent with national data [34]. While this analysis did not describe workforce within our centre, with the Irish National Cancer Strategy due for review in 2026/2027, these unique Irish data are a call to action for strategic development of the specialist cancer AHP workforce and service models to meet demands.

Across all five disciplines studied, *on treatment status* was consistently associated with higher unmet needs when adjusted for age, gender, cancer diagnosis, and cancer stage. Just over half of our sample (57%) were currently on treatment in the outpatient setting. These results suggest that cancer rehabilitation services need to consider models of care that support patients early in the treatment trajectory. Prospective surveillance models of care aim to embed planned impairment screening from diagnosis and at key timepoints through the treatment trajectory to identify impairment early and manage through evidence-based interventions [35]. This concept is reflected in prehabilitation-to-rehabilitation pathways which aim to enhance patient condition in advance of treatment, typically surgery, and provide planned postoperative rehabilitation to optimise outcomes [36]. These models of care are evidence-based, acceptable, and feasible to deliver clinically [37]; however, uptake and implementation internationally has been slow. Work is ongoing to examine the utility of triage and referral systems to support implementation of screening and referral through stepped care models of rehabilitation into practice [31]. While our analysis considered only hospital-based services, triage and referral systems recognise a spectrum of patient complexity arising from comorbidities or cancer/treatment-related side effects [38], and aim to direct patients to the right level of service to meet their needs, thus leveraging community-based services and reserving services in the acute sector for patients with the highest burden of need. Such models will be required to effectively and proactively manage rehabilitation needs in resource-limited healthcare systems.

Unsurprisingly, patients with a diagnosis of head and neck cancer were 4.97 times more likely to report speech and language therapy-specific needs and 3.34 times more likely to report dietetic-specific needs in adjusted models. Results were similar for patients with upper gastrointestinal cancer who were 3.77 times more likely to report speech and language therapy needs and 4.19 times more likely to report dietetic needs. Surgical resection for both head and neck, and upper gastrointestinal cancers can lead to transformative anatomical and physiological changes that result in long-term nutritional, speech, swallow, and quality of life sequelae [39–41]. Speech and language therapists are a highly skilled workforce who bring very specialised speech, swallow, and communication rehabilitation skills that are not available through other professions [7]; however, only 38% of our sample with identified speech and language therapy needs reported receiving speech and language therapy input since their diagnosis. There is a major need to proactively embed specialised AHPs into all aspects of cancer management, from clinical service delivery through to governance and policy structures, to build service capacity by influencing workforce planning, budget allocation, and models of care. This is particularly relevant to cancers with highly attritional and complex needs.

In this sample, younger patients were consistently more likely to report unmet needs relevant to medical social work, physiotherapy, and occupational therapy than older patients. This is consistent with international literature reporting that younger people are more likely to report unmet needs, particularly psychological concerns [42, 43]. Critically, in our sample, all patients under the age of 25 years reported at least one need relevant to medical social work. While patients aged <25 years represent a relatively low number of all cancers within our sample (5%) or indeed nationally (approximately 200 adolescent or young adult diagnoses/year in Ireland), the patient years-of-life impacted by loss of education, career, and financial progression or psychosocial development are significantly greater than adult-onset cancers [44]. The overall and cumulative burden of unmet needs relevant to medical social work was significant in this sample. While medical social workers may seek opportunities to leverage community services to support patients, consideration needs to be given to how models of care need to be tailored and adapted across the lifespan to address unique psychosocial, developmental, and financial challenges impacting different age groups [45].

In contrast, results demonstrating that younger age groups were more likely to report unmet physiotherapy and occupational therapy relevant needs were surprising and inconsistent with both clinical expectations and the wider literature. Using the Patient-Reported Outcome Measurement Information System–Physical Function (PROMIS-PF) assessment of physical functioning impairment, Flores et al. reported that as age increased in 5-year increments, patients were 19% more likely to have moderate–severe physical functional impairment [15]. In an analysis of 7495 patients aged >65 years with and without cancer, Gell et al. reported worsening scores with older age across a range of objectively completed performance-based measures [14]. While acknowledging that physiotherapy and occupational therapy roles in cancer extend beyond physical functioning [4, 5], it is a major domain. In this analysis, needs were self-reported as present or absent using the Macmillan HNA which does not quantify impairment severity or provide additional information about the domains of function that are impacted. Crude measures of perceived physical function can correlate poorly with objectively measured performance status in older adults with cancer [46] and therefore the results of our analysis should be interpreted with caution. Older adults have the highest cancer burden but are the age group most likely to be excluded from research [47]; consequently, the unmet needs of cancer survivors from aged >65 years are often inadequately managed [10].

This study has several strengths. The study sample is relatively large, diverse, and recruited over a short timeframe. Demographically, there is a good representation across cancer types and age ranges, which was achieved through prospective data entry informing a targeted recruitment strategy. The Macmillan HNA is clinically pragmatic, widely implemented, and can be easily mapped to clinical services. The work is delivered by a highly experienced interprofessional team of clinical and research AHP, nursing, and medical cancer specialists who informed the design and the analysis, in consultation with the TSJCI Patient Representative Group. There are, however, some limitations that warrant discussion. The Macmillan HNA is limited to cross-sectional, dichotomous answers and therefore does not provide a measure of severity or change over time which would inform whether the needs identified require community services (mild–moderate severity) or to acute AHP services (moderate-to-severe severity). Results signal areas that may benefit from further investigation with objective assessment protocols to deepen understanding of severity and associations of needs.

Furthermore, this paper focussed specifically on AHP service utilisation. Symptoms such as fatigue and psychological concerns may also be addressed by nursing and psychology colleagues which were not captured in this analysis. The professions examined align with international literature and were relevant to our local health system, and we acknowledge that the composition of AHP cancer rehabilitation teams may differ in other contexts and that the procedures used to map professions to concerns may have reduced validity outside of our setting. Our mapping procedures followed a very pragmatic, clinically informed approach which reflects practice and profession-specific roles within our Comprehensive Cancer Centre, but will require further testing to confirm its reproducibility and validity in other contexts. Patient-reported outcomes such as the Macmillan HNA are widely embedded within cancer survivorship with multiple advantages that support symptom control, communication, and quality of life [48]; however, challenges such as incomplete data, patient burden, and health literacy do exist. In implementing this study, efforts to optimise patient engagement and overcome challenges included providing the questionnaire in multiple languages, on-site research staff to answer questions, and providing the questionnaire in multiple formats. Interestingly, 96% of completed surveys were completed in paper format suggesting that this remains an important option in a digital world. Finally, patients self-reported cancer information and history of AHP access and therefore these data may be subject to recall bias and did not explore barriers to attending rehabilitation. Future research with larger funding could explore past and current service utilisation in more detail to determine the volume and timing of AHP input along the cancer trajectory, and barriers to service delivery.

In conclusion, results confirm that patient-reported rehabilitation needs are significant across the cancer trajectory, but that access to cancer rehabilitation AHPs is suboptimal. Strategic efforts to map current and future specialist workforce requirements for AHPs in cancer are urgently needed to inform future investment. This can only be achieved through AHP representation at all levels of cancer care and management, from clinical services through to policy. Without strategic development of cancer rehabilitation AHP workforce, patients will continue to unnecessarily experience functional, nutritional, and psychosocial impairments, with serious health-related quality of life and economic consequences [49, 50].

## Supplementary Information

Below is the link to the electronic supplementary material.

ESM1 (DOCX 39.5 KB)

## Data Availability

The datasets generated during and/or analysed during the current study are available from the corresponding author on reasonable request.
