# Supplementary material for: Unmet cancer rehabilitation needs and access to survivorship services across the cancer continuum
Source: Support Care Cancer. 2026 May 11;34(6):528. doi: 10.1007/s00520-026-10752-5 (PMC13160968; doi:10.1007/s00520-026-10752-5)
Supplement: Supplementary file 1 — (DOCX 39.5 KB) [file 520_2026_10752_MOESM1_ESM.docx]

**Unmet cancer rehabilitation needs and access to survivorship services across the cancer continuum**

**Supplemental File 1**

| **Profession** | **Domain** | **Needs** |
| --- | --- | --- |
| **Physiotherapy** | **Physical** | Breathing Difficulties |
|  |  | Cough |
|  |  | Swelling |
|  |  | Passing urine |
|  |  | Moving around (walking) |
|  |  | Pain or Discomfort |
|  |  | Tingling in Hands and Feet |
|  |  | Tired, Exhausted or Fatigued |
|  | **Emotional** | Independence |
|  | **Information or Support** | Exercise and Activity |
|  | | |
| **Occupational Therapy** | **Physical** | Breathing Difficulties |
|  |  | Moving around (walking) |
|  |  | Tingling in Hands and Feet |
|  |  | Tired, Exhausted or Fatigued |
|  |  | Memory or Concentration |
|  |  | Wound Care |
|  | **Practical** | Work or Education |
|  |  | Housing |
|  |  | Transport or Parking |
|  |  | Washing and Dressing |
|  |  | Preparing Meals and Drinks |
|  | **Emotional** | Independence |
|  | | |
| **Speech & Language Therapy** | **Physical** | Eating, Appetite, or Taste |
|  |  | Swallowing |
|  |  | Speech or Voice problems |
|  | **Practical** | Talking or Being Understood |
|  |  |  |
| **Dietetics** | **Physical** | Eating, Appetite, or Taste |
|  |  | Indigestion |
|  |  | Swallowing |
|  |  | Sore or Dry mouth or Ulcers |
|  |  | Constipation |
|  |  | Diarrhoea |
|  |  | Nausea or Vomiting |
|  |  | Tired, Exhausted or Fatigued |
|  |  | Wound Care |
|  |  | Changes in weight |
|  | **Practical** | Preparing Meals and Drinks |
|  |  | My Medications |
|  | **Information or Support** | Diet and Nutrition |
|  | | |
| **Medical Social Work** | **Practical** | Taking Care of Others |
|  |  | Work or Education |
|  |  | Money or Finance |
|  |  | Travel |
|  |  | Housing |
|  |  | Transport or Parking |
|  |  | Talking or Being Understood |
|  |  | Laundry or Housework |
|  |  | Grocery Shopping |
|  |  | Preparing Meals and Drinks |
|  |  | Difficulty making plan |
|  |  | Problems with Alcohol or Drugs |
|  | **Emotional** | Uncertainty |
|  |  | Loss of Interest in Activities |
|  |  | Unable to Express Feelings |
|  |  | Thinking of the Future |
|  |  | Regret about the past |
|  |  | Anger or Frustration |
|  |  | Loneliness or Isolation |
|  |  | Sadness or Depression |
|  |  | Hopelessness |
|  |  | Guilt |
|  |  | Worry, Fear or Anxiety |
|  |  | Independence |
|  | **Family or Relationship** | Partner |
|  |  | Children |
|  |  | Other Relative or Friends |
|  |  | Person who Looks after me |
|  |  | Person who I look After |
|  | **Spiritual** | Feeling at Odds with my Culture, Beliefs or Values |
|  | **Information or Support** | Planning for my Future Priorities |
|  |  | Making a Will or Legal Advice |
|  |  | Patient or Care Supports Groups |

**Supplemental File 1:** Mapped Allied Health Professional Expertise to Unmet Needs as Captured by the MacMillan Health Needs Assessment. Mapped completed by a team of clinical cancer rehabilitation specialists and underpinned by the literature **(1, 2, 3, 4, 5, 6, 7, 8, 9, 10, 11, 12, 13, 14, 15, 16, 17)**

.

**References**

1. MacMillan Cancer Support (2020) Cancer Rehabilitation Pathways Guidance. .

2. Macmillan Cancer Support (2017) The MacMillan Allied Health Professionals Competency Framework.

3. Cancer Cross Working Group. (2022). Report - The Role of Physiotherapy in Cancer Care in the Europe region. Europe Region of World Physiotherapy. <https://www.erwcpt.eu/cancer>

4. Pergolotti M, Williams GR, Campbell C, Munoz LA, Muss HB. Occupational Therapy for Adults With Cancer: Why It Matters. Oncologist. 2016;21(3):314-9.

5. Kelly L, Datta M, Arthur AE. Revised 2025 Scope and Standards of Practice for Registered Dietitian Nutritionists in Oncology Nutrition. Commission on Dietetic Registration. <www.cdrnet.org/focus>. .

6. Rothrie S, Fitzgerald E, Brady GC, Roe JWG. The role of the speech and language therapist in the rehabilitation of speech, swallowing, voice and trismus in people diagnosed with head and neck cancer. Br Dent J. 2022;233(9):801-5.

7. Zebrack B, Zhang A, Ghazal LV, Francis-Levin N, Brandon RE. The Essential Nature of Social Work in Cancer Control. Cancer Control. 2025;32:10732748251353081.

8. Gillman A, Kenny C, Hayes M, Walshe M, Reynolds JV, Regan J. Nature, severity, and impact of chronic oropharyngeal dysphagia following curative resection for esophageal cancer: a cross-sectional study. Dis Esophagus. 2024;37(5).

9. Kenny C, Regan J, Balding L, Higgins S, O'Leary N, Kelleher F, et al. Dysphagia Prevalence and Predictors in Cancers Outside the Head, Neck, and Upper Gastrointestinal Tract. J Pain Symptom Manage. 2019;58(6):949-58.e2.

10. National Institute for Health and Care Excellence: Guidelines. Cancer of the upper aerodigestive tract: assessment and management in people aged 16 and over. London: National Institute for Health and Care Excellence (NICE)

Copyright © NICE 2019.; 2018.

11. National Institute for Clinical Excellence. (2004). Improving outcomes in head and neck cancers: Cancer service guideline (CSG6). <https://www.nice.org.uk/>.

12. EFAD &amp; ESDN Oncology Statement Paper on the Role of the Dietitian in Oncology. Kompass Nutrition & Dietetics. 2021;1(3):117-.

13. Berger MM, Shenkin A, Schweinlin A, Amrein K, Augsburger M, Biesalski HK, et al. ESPEN micronutrient guideline. Clin Nutr. 2022;41(6):1357-424.

14. Arends J, Bachmann P, Baracos V, Barthelemy N, Bertz H, Bozzetti F, et al. ESPEN guidelines on nutrition in cancer patients. Clin Nutr. 2017;36(1):11-48.

15. Arends J, Baracos V, Bertz H, Bozzetti F, Calder PC, Deutz NEP, et al. ESPEN expert group recommendations for action against cancer-related malnutrition. Clin Nutr. 2017;36(5):1187-96.

16. Mountford CG, Manas DM, Thompson NP. A practical approach to the management of high-output stoma. Frontline Gastroenterol. 2014;5(3):203-7.

17. Greally, H., Love, D., & Mullen, L. (2020). Hospital and Community-based Psychosocial Care for patients with cancer and their families: A Model of Care for Psycho-Oncology. National Cancer Control Programme: Dublin. .
